# Effects of Reflection to Improve Goal-Directed Self-Talk on Endurance Performance

**DOI:** 10.3390/sports6020055

**Published:** 2018-06-13

**Authors:** Alexander T. Latinjak, Bernat de las Heras, Arnau Sacot, David Fernandez, Daniel Robinson, Andrew M. Lane

**Affiliations:** 1 Department of Science and Technology, University of Suffolk, IP3 0FS Ipswich, UK; 2 Escola Universitària de la Salut i l’Esport, University of Girona, 17190 Salt, Spain; dfergon4@gmail.com; 3 School of Physical & Occupational Therapy, McGill University, Montreal, QC H3G 1Y5, Canada; bernat.delasheras@mail.mcgill.ca; 4 School of Sport and Exercise Sciences, John Moores University, L3 5UA Liverpool, UK; arnau.sacot@gmail.com; 5 Faculty of Education, Health and Well-Being, University of Wolverhampton, WS1 3BD Walsall, UK; therunningdan1979@gmail.com (D.R.); A.M.Lane2@wlv.ac.uk (A.M.L.)

**Keywords:** self-talk, effort, perceived exertion, endurance, psychological skills

## Abstract

We investigated the effects of an intervention that encouraged reflection on organic self-talk used during endurance performance. Using an experimental design, we compared the effects of enhancing metacognitive skills by (a) planning and (b) reviewing and evaluating goal-directed self-talk. Participants completed three time-to-exhaustion cycling task trials in which we hypothesized that the intervention group would perform significantly better than the control group. Further, we expected a reduction in perceived exertion for a given workload among participants following a self-talk intervention. Thirty-four participants completed a time-to-exhaustion cycle ergometer test, after which participants were randomly divided into an intervention and control group. The intervention group performed reflection tasks on performance in the time-to-exhaustion test. Participants completed two further time-to-exhaustion tests. Repeated measures analyses of covariance to test whether the intervention group performed for longer indicated no significant difference in time to exhaustion (*p* = 0.157). Perceived exertion rates were 2.42% higher in the intervention compared to the control group (*p* = 0.025). In conclusion, in the intervention group, goal-directed self-talk led to increased sensitisation to perceived exertion, and participants chose to stop exercising at this point rather than repeat implementation of self-talk statements and persist for longer.

## 1. Introduction

Endurance performance is a fundamental requirement of many sports and can be defined as the ability to withstand stress over a prolonged period. Research on the physiological basis of endurance performance is undecided as to what causes fatigue and ultimately a decline in performance [1]. Given the difficulty of identifying isolated physiological factors, research has highlighted the contribution of psychological factors and the potential benefits of psychological skill training [2]. Psychological qualities such as improved confidence can be developed via skills training [3]. Skills found to be effective in improving endurance performance include cognitively based approaches such as the use of imagery, self-talk, and goal setting [4,5,6]. In the present study, we focus specifically on self-talk, which can be defined as an act of syntactically recognizable communication [7], articulated either out loud or as a voice inside the head [8], addressed to the self, with interpretative elements associated to its content [9].

### 1.1. Self-Talk Interventions

Self-talk interventions appear to have positive effects on endurance performance in running, cycling, and swimming [10,11,12,13,14,15]. Some of the differences between studies on self-talk interventions in endurance contexts are of interest as they offer practitioners flexibility in designing and implementing self-talk to adapt the intervention in applied contexts. Of particular relevance is the degree to which the narrative used in the self-talk intervention is predetermined by the researchers or self-determined by the athletes [9]. For example, Hatzigeorgiadis et al. [12] gave swimmers cue words to use along with instruction as to when and how to use them. Barwood et al. [10] used recreationally active participants and provided two motivational self-statements (e.g., I can manage my energy until the end), which they rehearsed in the days preceding and immediately before the final 10-km cycling time-trial. A consistent feature of interventions is that they are (a) directed towards the mechanisms through which self-talk aids performance (e.g., self-talk is motivational) and (b) strongly advise that cues are used during task execution. Regarding the latter, manipulation or compliance checks are frequently used to monitor whether participants used their cues or whether they used different cues [14]. Notwithstanding, (a) and (b) do not apply in the case of the most self-determined forms of self-talk interventions.

Not all self-talk interventions described in the literature require their participants to use the cues previously established. Some interventions are solely targeted at making the athletes reflect on their past self-talk to explore alternative cues for future practice [16,17]. The aim of these interventions is to improve the quality of athletes’ goal-directed self-talk, that is, statements deliberately employed towards solving a problem or making progress on a task [18]. To date, the only example for such a self-talk intervention is a single-case study describing a goal-directed self-talk intervention with a 36-year-old elite orienteer [13]. The goal-directed self-talk intervention comprised questioning original organic self-talk and theoretically exploring alternative instructions, leaving it open for the athlete to decide when to put them into practice. Providing preliminary idiosyncratic evidence, the study suggests that the intervention is closely related to the improvement of metacognition, as the athlete indicated that “my evaluation about the intervention is very positive thanks to our analysis, understanding, and application of self-talk… Without this understanding of the situations, their causes and effects, successful application of self-talk is highly unlikely” [13] (p. 193). Offering further indirect support for the application of a goal-directed self-talk intervention to endurance performance, studies on cognitive components in endurance tasks have demonstrated that metacognition is an essential component of self-regulation and its primary functions are to monitor and control the thoughts and actions required for endurance task completion [19].

### 1.2. The Present Study

Overall self-talk interventions based on the use of predetermined or self-determined cues [15] have shown to be performance-enhancing in endurance tasks. However, there is a dearth of research on the effects of goal-directed self-talk interventions [13], which consist of planning, reviewing, and evaluating goal-directed self-talk on performance in endurance tasks. To provide experimental evidence on the effects of goal-directed self-talk interventions in endurance tasks, this study aimed to analyse the effects of enhancing metacognitive skills related to goal-directed self-talk by (a) planning and (b) reviewing and evaluating three trials of a time-to-exhaustion cycling task on performance (i.e., time to exhaustion) and rates of perceived exertion. With regard to the specific hypotheses, we expected the intervention group to improve over time. Additionally, if the control group improved as well, we expected the improvement of the intervention group to be greater. The improvement of the control group would be explained by a learning effect from repeating the same task.

## 2. Method

### 2.1. Research Design

The study was a repeated-measures design in which participants made four visits to the laboratory. Participants were randomly split into either a control or intervention group.

### 2.2. Participants

Athletes (*N* = 220) were approached at a sport science faculty before and after their regular lectures. A total of 34 athletes (*M*_age_ = 21.56, *SD* = 2.27) volunteered for the study—14 female athletes and 20 male athletes (see Table 1). They were all practicing and competing in sport regularly (7.17 h/week, *SD* = 3.34). Seven athletes withdrew from the experiment (five from the control group and two from the experimental group) due to injuries which were unrelated to the experimental task (*n* = 3), time constraints (*n* = 3), and lack of motivation (*n* = 1). Finally, 12 participants completed the four sessions in the control condition (5 females and 7 males) and 15 participants completed the four sessions in the intervention condition (6 females and 9 males).

### 2.3. Time-to-Exhaustion Tests

#### 2.3.1. Incremental Test to Exhaustion for Peak Power Output

**Figure 1:**
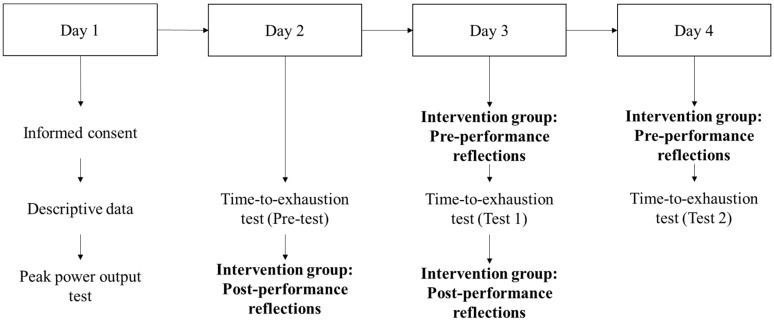
Experimental design diagram. Note. Bold sections indicate the different parts of the goal-directed self-talk intervention.

Participants’ maximal workload was determined by means of a progressive and maximum aerobic test [20] consisting of an initial load of 20 W followed by increases of 20 W min^−1^ until participants could not maintain the required cadence (70 rpm) for 10 consecutive seconds (see Figure 1). For results on the peak power output, see Table 1.

#### 2.3.2. Cycle Endurance Test

Following the procedures in Blanchfield et al. [10], the time-to-exhaustion test commenced with a 3-min warm up at 40% of the participants peak power output. After 3 min, the power output was automatically increased to a power output corresponding to 80% peak power output (PPO). Cadence was set at 70 RPM. Time to exhaustion was defined as the time accrued from the onset of the 80% PPO until the point at which cadence had fallen below 70 RPM for 10 consecutive seconds. No verbal encouragement was provided at any point during the time-to-exhaustion test.

### 2.4. The Goal-Directed Self-Talk Intervention

The intervention started immediately after the data collection in Session 2 (see Figure 1). The intervention consisted of post-performance reflections used to identify what goal-directed self-talk was used during the cycle endurance tests. Participants were asked to anticipate possible issues and consider goal-directed statements which could be used to help cope with hypothetical problems in following trials (Test 1 and Test 2).

In contrast, the control group was not asked to perform any self-reflection. This decision is in line with previous studies in self-talk in which participants of the control groups were asked to keep performing as usual [10]. Because athletes who participate in goal-directed self-talk interventions are not asked to use any of their previously discussed statements during task execution, this study did not contemplate the use of manipulation or compliance checks.

#### Self-Generated Self-Talk Intervention Developed via Reflection

We used self-reflection to develop a self-generated, self-talk intervention. Participants were asked to reflect on experiences before and during performance. The aim of the booklet was to encourage self-reflection via recollection of intense experiences that they considered were negative and undesirable. We specified that people often report unpleasant feelings in pursuit of a goal and if they achieved the goal and appraised these feelings as a necessary part of performance management, then they were not considered negative and undesirable. For example, an individual could respond that she performed badly because she felt fatigued and wanted to slow down, which prompted anger and frustration as she would not achieve her goal, a goal that was publicly known to her significant others. Using this negative experience, participants were encouraged to consider self-talk solutions, and so using the present example, the participant could have reappraised feelings of fatigue as a necessary part of goal attainment and altered her focus to task-relevant cues.

To promote further reflection and help participants identify which self-talk statements were helpful and which were not, athletes were asked to rate each goal-directed statement reported in the earlier section with two 5-point Likert scales. Specifically, they were asked to rate how frequently they had used that self-statement on a scale ranging from 1 (only once) to 5 (permanently), and to what degree they believe the statement had helped them improve performance from 1 (completely irrelevant) to 5 (helped decisively).

Self-reflection was also focused on pre-performance thoughts by asking participants to anticipate problematic situations. A problematic situation would consist of any thought (e.g., disengagement thoughts), emotion (e.g., dejection), or physical sensation (e.g., fatigue) that impairs performance. Second, for each problematic thought, emotion, or physical sensation, the participants were asked to elaborate as many as three possible goal-directed self-statements they could use to solve the problem or make progress on the task.

Given that the aim of the reflective process was to develop self-generated interventions, participants were asked to think about potential alternative goal-directed statements which could be useful.

### 2.5. Procedure

Permission to carry out the study was granted from the local ethics committee, and all participants signed the informed consent form at the beginning of the first session. The study was conducted in accordance with the Declaration of Helsinki. Participants were informed of the task requirement but remained blind to its specific purposes. Before each session, they were explicitly briefed in the procedures of the session and informed that they could withdraw from the session at any time without giving any reason and with no negative consequences.

All sessions were individual meetings between one researcher and the participant to control for social factors [21]. Sessions were conducted at least two days apart to ensure sufficient recovery [22]. Cycling tasks were performed on a cycle ergometer (Ergoselect 100, Ergoline, Bitz, Germany) with saddle and handlebar specifications adjusted to fit the preference of each participant using guidelines by Balagué et al. [23], and these measurements remained identical for all experimental sessions.

All tests were conducted between 8:30 a.m. and 6:00 p.m. on the same cycle ergometer with the screen covered to avoid seeing any data output. Participants performed all tests at approximately the same period of the day. All trials were video recorded to verify the obtained data and check for possible errors in their collection, and heart rate was continuously monitored (Polar H7). Once the test began, to prevent bias from audience effects upon self-talk measurements, experimenters stood outside the participant’s angle of view, and participants were not exposed to any verbal or other communication.

Before each data collection, they were asked to complete a sport behaviour, rest, and drink checklist [24]. Participants were asked to confirm that they had not taken part in any heavy exercise in the 24 h prior to testing and refrained from the consumption of caffeine and nicotine in the 3-h period leading up to each test. If a participant had done any of these three things, the session was interrupted and rescheduled. Finally, after each session, participants were given a sandwich and a drink to aid recovery and as a sign of gratitude for their help. Researchers who conducted the session were trained in the data collection procedures but remained blind during the data collection to the specific purposes of the study.

### 2.6. Measures

Three dependent variables were analysed in this study: time to exhaustion, maximum heart rate, and rates of perceived exertion (RPE). With regard to the latter, upon task completion, the RPE 6-to-20 scale [25] was placed in front of participants in order to assess RPE. In previous studies, RPE was recorded at 1-min intervals [10]. However, in pilot trials, participants perceived that reporting RPE each minute drew their attention to fatigue and away from self-talk. To avoid interference with participants’ thoughts and self-talk during the task, we assessed RPE after each trial.

### 2.7. Data Analyses

To test for differences between both groups prior to the intervention, we ran independent-samples *t*-tests for (a) participants’ descriptive data (i.e., age, weight, height, and PPO) and (b) performance variables (i.e., time to exhaustion, maximum heart rate, and RPE) in Session 2. To test our hypotheses, we performed 2 (groups) × 2 (intervention trials) repeated measures analyses of covariance (RM-ANCOVA), using scores in the pre-test on Day 2 as covariables. We examined the group, time, and group × time effects on time to exhaustion, maximum heart rate, and RPE. Effect size was calculated for all significant effects. Based on the criteria outlined by Cohen [26], thresholds for small, moderate, or large effect sizes were set at 0.1, 0.3, and 0.5, respectively [27].

## 3. Results

### 3.1. Preintervention Comparisons between Groups

The comparisons between both groups in terms of participants’ descriptive data and performance variables on Day 2 yielded no significant differences prior to the intervention (all *p >* 0.05). Descriptive data for participants’ descriptive data showed that participants weighed between 51 and 95 kg (*M* = 69.04, *SD* = 11.11) and measured between 158 and 195 cm in height (*M* = 174.96, *SD* = 9.48). Furthermore, peak power output ranged between 180 and 360 W (*M* = 253.33, *SD* = 54.07) and maximum heart rate during the PPO test ranged between 153 and 198 heartbeats/min (*M* = 182.89, *SD* = 11.80).

### 3.2. Main Analyses

As Table 2 indicates, RM-ANCOVA results for time to exhaustion, maximal heart rate, and RPE indicated a significant group effect for RPE, whereby results show the intervention group reported higher RPE compared to the control group (*F*_1,24_ = 5.72, *p* = 0.025, *η*^2^ = 0.192). There were no other significant interaction or main effects over time. Effect size was nonetheless small. For descriptive data and all RM-ANCOVA statistics, see Table 2.

### 3.3. Self-Talk Frequency and Perceived Efficacy in the Intervention Group

An RM-ANOVA yielded a significant time effect on self-talk frequency (*F*_2,28_ = 7.07, *p* = 0.003, *η*^2^ = 0.336), indicating that frequency of self-talk was higher in Test 2 (*M* = 3.65; *SD* = 0.71) compared to the pre-test (*M* = 3.22; *SD* = 0.60) and Test 1 (*M* = 3.30; *SD* = 0.62). Time effects on perceived efficacy were not significant (*F*_2,28_ = 0.49, *p* = 0.619), indicating that there were no significant changes from pre-test (*M* = 3.68; *SD* = 0.48) to Test 1 (*M* = 3.57; *SD* = 0.60) and Test 2 (*M* = 3.54; *SD* = 0.58).

## 4. Discussion

Our research was predicated on the notion that self-reflection on performance would help develop an effective narrative for self-talk. Previous evidence has supported positive effects of motivational self-talk interventions on endurance performance [10,11,15] and the effects of goal-directed self-talk interventions in other sport settings [13]. An experiment was established to test the extent to which engaging with formal self-reflection associated with better performance and that this could be explained by reduced physiological load and lower perceived exertion. In contrast to these predictions, our results indicate that following a reflection-based intervention did not associate with improved performance or a reduced heart rate. However, results did show that self-reflection training associated with an increase in ratings of perceived exertion. We offer two explanations for these results: (a) self-talk as an intervention tool may not be useful in this context, and (b) the approach followed to teaching self-talk via reflective experiences may have been ineffective; that is, how people learn to use self-talk requires further examination. The fact that participants in the intervention group reported increased frequency of self-talk use but no changes in perceived self-talk efficacy is indirect endorsement that the reflection techniques were ineffective in the context of the endurance task.

The nature of the reflective tasks helped participants become conscious of negative experiences and help develop individualized self-talk scripts. Previous research has found such an approach has desirable benefits as athletes develop greater ownership of their interventions [16]. By linking the self-talk to an experience where it was successful and strengthening this association through practice, its usage is proposed to be enhanced. However, such a strategy relies on knowledge of how long the person needs to endure the intense fatigue and be able to use self-talk to overcome these effects [6]. Evidence shows people find increased energy, demonstrated behaviourally, by increasing speed when the finish line is anticipated [28]. People ration the resources needed to complete against perceived available resources, or the resources they are prepared to give, and use strategies to activate personal resources to bridge the gap [29]. In this example, people have clear reference points on which to use self-regulated strategies to manage fatigue. In the present study, the intervention encouraged reflection on coping with fatigue but not fatigue in relation to a specific outcome goal. The absence of performance feedback during the task meant that participants could not develop schema on how they could use feedback from performance to gauge how much effort to use. Self-talk can raise emotional arousal which can lead to improved performance via increased effort [4]. Further, research has indicated that it is possible to learn self-talk to raise arousal using brief interventions [4]. In the present study, by blinding participants from feedback, we also blinded them from the opportunity to use self-talk at the point when it would have been useful. Beedie et al. [28] showed that when feedback was withheld, riders reported experiencing negative internal thoughts and emotions. Further, riders reported the same intensity of exercise was harder than when full feedback was offered. It is possible that participants started to feel fatigue and interpreted this by expecting to stop, particularly as they had done the test previously and so this experience could be used as a reference point.

In the present study, with a task where the intensity was set at the start of exercise and at 80% peak power, it was intentionally difficult from the outset so that physiological cues would pre-emanate [30] and participants would have the option to repeat usage of self-talk. Arguably, by raising awareness of self-talk as an intervention strategy, this could have prompted earlier and repeated usage. If self-talk is raising arousal, then this is not a strategy that is necessarily useful in endurance sports, as the sustainability of intense arousal will be challenging unless the goal being pursued is considered highly valuable [6]. Recent research has found that using strategies such as self-talk at opportune moments is desirable [14]. Presenting self-talk statements in an if-then format, where the “if” part represents the challenge (“If” I have thoughts that the ride is too hard) and the “then” part represents the solution (“then I will tell myself, ‘push myself, you can do another 60!’”), represents a worthwhile approach to follow in future research.

In the present study, we expected there to be differences in performances between the intervention and control group, such as one group improving more than the other. However, neither group significantly improved over time. Given the intense nature of the task and that participants were not experienced cyclists, it is possible to suggest that performance in the control group could have deteriorated over time. Although the control group did not receive the training package, we posit that while repeating a new task, organic self-regulation skills are adjusted to meet the specific effort demands placed by the task [31]. In this case, the participants knew they had to repeat the task on Day 3 and 4. To do so, it seems likely that they remembered their struggles and thought about how to cope with them in the future. Consequently, participants in both groups engaged in some reflection and planning, which are fundamental to create metacognitive knowledge [32] and also underlie the mechanisms of the intervention tested in this study. Hence, the degree to which the intervention could have made a difference in the participants’ self-regulation was confounded with the effects of repeating the task over time. For future research, we would therefore suggest using either longer baselines or previously known tasks. In both cases, participants would have achieved a ceiling effect in their self-regulation behaviours, and the intervention could have prompted noticeable changes in their goal-directed self-talk.

Second, the intervention offered too few opportunities for the participants to switch from an obvious, yet ineffective, self-talk approach (i.e., goal-directed self-talk aimed at increasing effort) to an alternative, more effective, approach (e.g., goal-directed self-talk aimed at distracting from fatigue). Little is known about the learning process by which athletes improve their organic, goal-directed self-talk. More specifically, we do not know about the success/failure rate required for the participants to challenge their initial self-talk approach and switch to alternative approaches and test their effectiveness. From the participants’ reflection booklets, we know that the type of self-talk used remained largely unchanged throughout the three sessions. We suggest that future studies should use longer interventions to give participants time to challenge their initial approach to self-talk and to become aware that alternative approaches to self-talk might be more helpful. For instance, we suggest that goal-directed self-talk, distracting the participant from fatigue during the early stages of the task, would have been more helpful. Participants would have anticipated lower fatigue and therefore been able to persist for longer [33]. However, such an assumption is based upon participants being able to use strategies to persist for longer and with no feedback on how long they were exercising.

### Limitations

With regard to the limitations of this study, several aspects which require consideration have been listed in the preceding paragraphs. Furthermore, it could be argued that a larger number of participants was required or that the intervention was too short to produce noticeable effects. Similar numbers of participants allowed previous studies to evidence beneficial effects of self-talk on time-to-exhaustion performance (i.e., *N* = 24, [11]; *N* = 18, [15]). Seven participants withdrew from the experiment. These participants belonged mainly (*n* = 5) to the control group. Yet, considering the causes for withdrawal, it would seem incoherent to draw any conclusion upon the experimental conditions. Finally, considering the length of the intervention (range: 17–21 days from Session 2 to 4), it could be argued that additional sessions would have elicited more beneficial effects from the goal-directed self-talk intervention.

## 5. Conclusions

This study is the first to test the effects of a goal-directed self-talk intervention, consisting of analysing and reflecting upon goal-directed self-talk used during a time-to-exhaustion endurance task. Our results do not support the intervention. Nonetheless, we regard these negative results as equally significant as they indicate that our current conceptual understanding of self-talk interventions is incomplete. Furthermore, we also consider that negative results are relevant per se for the self-talk literature, as their absence from the literature would inflate effect size estimates in future meta-analyses, thus exaggerating the importance of self-talk interventions [34]. Accordingly, results that do not confirm expectations, such as ours, are crucial to scientific progress which is only made possible by a collective self-correcting process.

## Figures and Tables

**Table 1:** Descriptive data for age, weight, height, and peak power output for participants in the control and intervention groups.

Descriptors	Control Group		Intervention Group	
Age (years)	*M =* 22.00	*SD* = 2.83	*M =* 21.20	*SD* = 1.86
Weight (kg)	*M =* 68.00	*SD* = 11.72	*M =* 69.87	*SD* = 10.95
Height (cm)	*M =* 172.17	*SD* = 9.91	*M =* 177.20	*SD* = 8.82
Peak power output (W)	*M =* 251.67	*SD* = 63.51	*M =* 254.67	*SD* = 47.49

**Table 2:** Descriptive data and analyses of variance for performance maximal heart rate and rates of perceived exertion for all participants in the pre-test and for each group in the following two tests.

Group/Test	Time to Exhaustion		Maximal Heart Rate		RPE (6–20)	
Pre-test	*M =* 589.59	*SD* = 209.48	*M =* 178.89	*SD* = 11.18	*M =* 16.89	*SD* = 1.95
Control group						
Test 1	*M =* 646.88	*SD* = 377.84	*M =* 176.83	*SD* = 10.96	*M =* 16.83	*SD* = 2.12
Test 2	*M =* 582.88	*SD* = 336.18	*M =* 170.42	*SD* = 12.19	*M =* 16.92	*SD* = 2.19
Intervention group						
Test 1	*M =* 582.60	*SD* = 186.23	*M =* 177.13	*SD* = 10.88	*M =* 17.40	*SD* = 1.50
Test 2	*M =* 579.60	*SD* = 208.65	*M =* 175.93	*SD* = 10.11	*M =* 17.33	*SD* = 1.35
RM-ANCOVA ^1^						
Time effect	*F =* 0.34	*p* = 0.566	*F =* 0.02	*p* = 0.886	*F =* 0.19	*p* = 0.667
Group effect	*F =* 2.98	*p* = 0.100	*F =* 0.91	*p* = 0.349	*F =* 5.72	*p* = 0.025
Interaction effect	*F =* 2.16	*p* = 0.157	*F =* 3.34	*p* = 0.080	*F =* 0.13	*p* = 0.725

**^1^** Pre-test scores were set as covariable for the 2 (Groups) by 2 (Tests) repeated measures analysis of variance.
